# Left Ventricular Myxoma: A Case Report

**DOI:** 10.7759/cureus.46112

**Published:** 2023-09-28

**Authors:** Diya Asad, Mass A Abu Moch, Husam Amarin, Ahmad W Abdeen, Jenan Sinokrot, Nouraldin M Bhais, Yasmin N Arda, Omar R Khalil, Tammah Safadi, Hasan Khatib

**Affiliations:** 1 Faculty of Medicine, Al-Quds University, Jerusalem, PSE; 2 Internal Medicine, Al-Quds University, Jerusalem, PSE; 3 Cardiac Surgery, Al-Makassed Hospital, Jerusalem, PSE

**Keywords:** echocardiography, median sternotomy, surgical removal, cardiac myxoma, left ventricle

## Abstract

Cardiac myxomas are the most common primary cardiac neoplasms, with only a small percentage being found in the left ventricle. Herein, we describe a 25-year-old male who presented with a complaint of chest pain for almost three months and was found to have a 2x2 cm encapsulated tumor attached by a 2-3 mm stalk to the mid-septum, 5 cm below the aortic annulus, via echocardiography. Additionally, a chest CT angiography was performed and revealed a small defect in the left ventricle with a low attenuation density originating from the septum. The tumor was later managed surgically with a median sternotomy approach, and left ventricular myxoma was confirmed histopathologically. Even though cardiac myxomas are incredibly uncommon, they are usually located in the left and right atria and are very unlikely to present in the left ventricle. This incident highlights the importance of taking cardiac myxoma into account as a potential differential diagnosis in cases of chest pain to prevent any further complications.

## Introduction

Cardiac myxomas are oval or round-shaped, mobile, pedunculated intra-cardiac neoplasms [1]. They are the most common primary cardiac tumor, with an incidence of up to 1 per million individuals [2]. About 75% of myxomas are encountered as single left atrial tumors, 20% are found in the right atrium, and only 2-3% are located in the left ventricle [3]. The majority of cardiac myxomas are sporadic, but they can also be familial in certain cases [2].

Cardiac myxomas are histologically benign but may be as worrying as a malignant tumor because they may lead to life-threatening complications such as hemodynamic disturbances or systemic embolization. The manifestations of cardiac myxomas depend on their location, size, and mobility. However, they can result in the classic triad of obstructive, embolic, and constitutional symptoms [2]. In this paper, we present a rare case of left ventricular myxoma that was diagnosed using echocardiography, treated surgically, and confirmed pathologically.

## Case presentation

A 25-year-old male patient visited the cardiology outpatient clinic, reporting vague chest discomfort persisting for approximately three months. Physical examination revealed normal heart sounds without any murmurs, and there was no reported history of syncope, palpitations, dyspnea, edema, or other signs of heart failure. Blood pressure, heart rate, and other vital signs were also normal. His past medical history was significant for a previous diagnosis of gastritis, for which he received antibiotics for a positive Helicobacter pylori (H. pylori) stool antigen without improvement. An initial ECG showed no signs of ischemic changes or any other abnormalities, and the chest pain was initially attributed to gastritis. Despite this, as his symptoms persisted, he underwent an exercise stress test, which yielded normal results and ruled out an ischemic origin of his pain. Subsequently, due to the persistent and unexplained nature of his chest pain, he underwent an echocardiogram.

**Figure 1:**
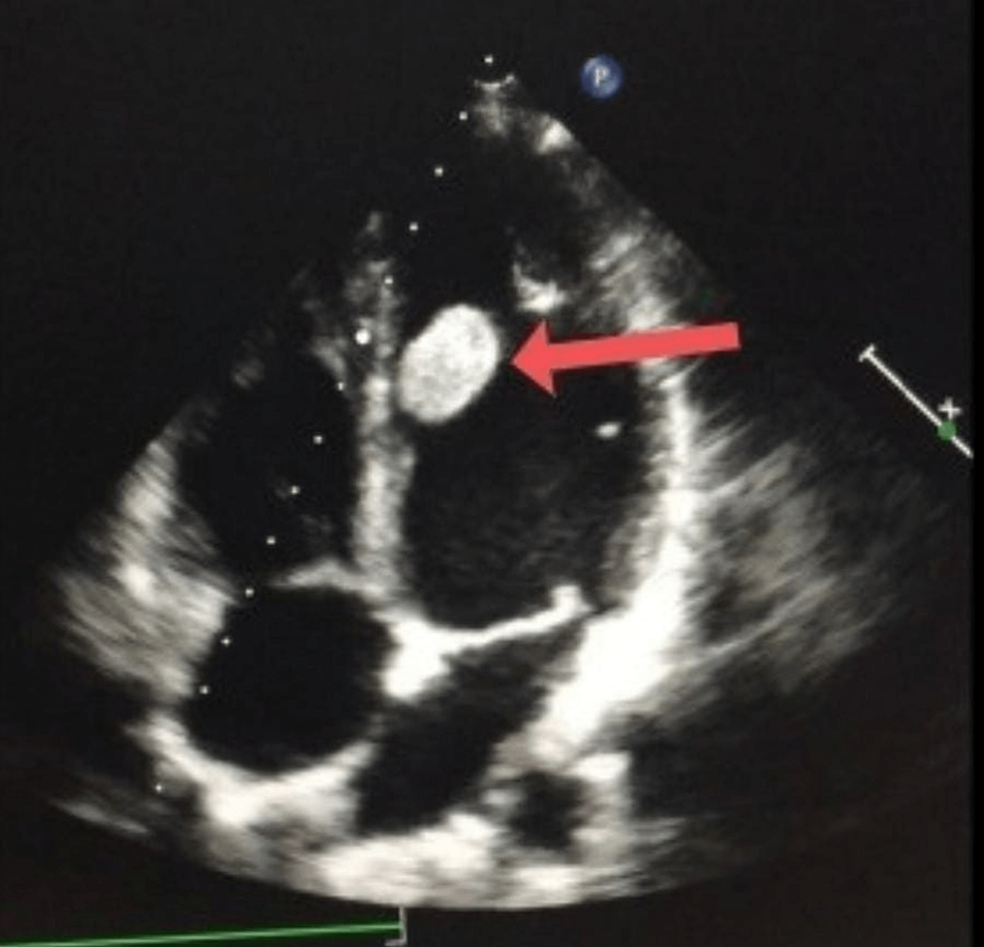
Echocardiography showing a 2x2 cm encapsulated tumor attached to the mid-septum below the aortic annulus.

Echocardiography showed a 2x2 cm encapsulated tumor attached by a 2-3 mm stalk to the mid-septum, 5 cm below the aortic annulus (Figure 1). Other parameters obtained from the echocardiogram include a left ventricular end-diastolic diameter (LVEDD) of 4.1 cm, a left ventricular end-systolic diameter (LVESD) of 2.7 cm, and a left ventricular ejection fraction (LVEF) of 60%. CT angiography of the chest revealed a small defect in the left ventricle (LV) measuring 2x1.3 cm, with a low attenuation density originating from the septum.

The surgical approach was performed by median sternotomy, aorto-bicaval cardiopulmonary bypass, and normothermic anterograde blood cardioplegia. After the hockey stick aortotomy, a translucent fragile jelly-like tumor was visualized in the left ventricle through the aortic cusps, which was attached to the septum by a stalk. The left atrial vent was inserted in the LV through the right ventricular systolic pressure (RSVP) under vision.

**Figure 2:**
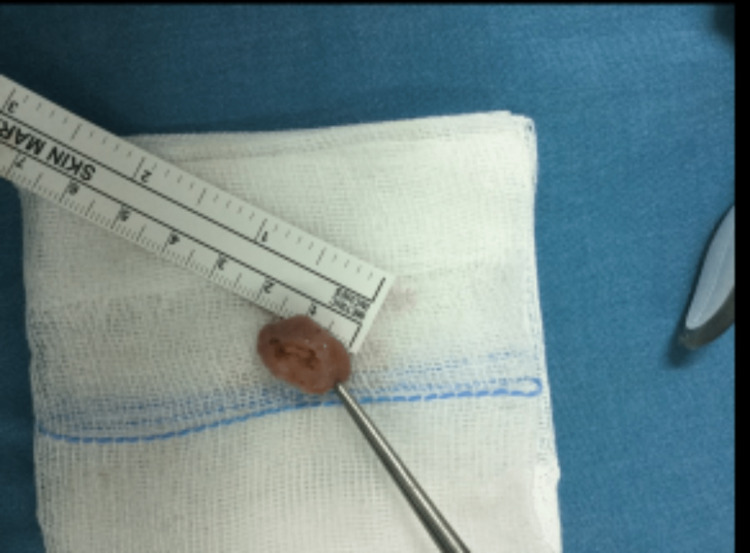
Cardiac tumor removed from the left ventricle

The stalk was cut with a harmonic scalpel, and the tumor was removed entirely en bloc (Figure 2).

The postoperative course was uneventful. The patient was discharged home on the fourth postoperative day. Histopathological examination was consistent with the diagnosis of a cardiac myxoma. We followed up with the patient for six months, and he reported a complete resolution of his symptoms with no recurrence of the myxoma. Plasma Interleukin could not be measured in this case.

## Discussion

Primary cardiac tumors are extremely rare. Cardiac myxoma is the most common type, accounting for 30-50% of all primary cardiac tumors. According to recent studies, the left ventricle accounts for only 2-3% of cardiac myxomas. Even though cardiac myxomas have a benign histology, they may result in serious outcomes. Due to their fragmentability, these tumors may worsen existing problems or potentially cause abrupt death. Myxoma of the LV causes a variety of cardiac symptoms such as syncope, dyspnea, embolic events, arrhythmias, or hemodynamic compromise [4,5]. Additionally, interleukin, which may cause inflammatory or autoimmune issues, is produced and released into the bloodstream by cardiac myxomas [6].

The gold standard method for confirming the diagnosis of myxoma is pathology. Cardiac myxomas often manifest as a single, fragile, irregularly shaped lesion that is pedunculated and has an intact capsule [7]. Histologically, they are characterized by irregular or star-shaped cells that are radially scattered around tiny blood arteries and loosely dispersed within a mucoid ground substance [7]. The best method for early myxoma detection is echocardiography, which is noninvasive and reliable. It allows for a quick and simple evaluation of the morphology, extent, site of attachment, involvement of valve leaflets, and functional obstruction of the LV outflow tract [4]. Myxomas have been reported to grow at a rate of 0.15 cm each month, so even if there were no symptoms of obstruction or embolism, surgical excision should be done as soon as the diagnosis is established [5].

The location of left ventricular myxomas determines the type of surgical incision to be made [4]. In our case, the myxoma was attached by a 2-3 mm stalk to the mid-septum 5 cm below the aortic annulus, necessitating a median sternotomy.

When the tumor is removed, the risk of fragmentation and the emergence of embolic phenomena are reduced by careful management of the cardiac structures and the tumor. To prevent recurrence, the resection of the tumor's base should be carried out with good safety margins [4].

A high rate of long-term survival and a favorable surgical prognosis are characteristics of cardiac myxomas. To assess for myxoma recurrence, routine postoperative echocardiographic evaluation is necessary [7].

## Conclusions

In conclusion, cardiac myxoma is the most common primary tumor of the heart. Left ventricular myxoma is an incredibly rare tumor. Echocardiography is the most effective approach for detecting early myxoma and should be considered early in the diagnosis to avoid life-threatening complications. The use of echocardiography, CT angiography, and histopathological evaluation in combination may aid in making an accurate diagnosis. Surgical excision should be performed as soon as the diagnosis is confirmed, and the type of surgical incision is based on the location of the cardiac myxoma. Our case report strives to increase understanding of cardiac myxomas, particularly those located in the left ventricle, and offers insight for future clinical management and research studies.
